# Multidrug resistance protein MdtM adds to the repertoire of antiporters involved in alkaline pH homeostasis in *Escherichia coli*

**DOI:** 10.1186/1471-2180-13-113

**Published:** 2013-05-23

**Authors:** Scarlett R Holdsworth, Christopher J Law

**Affiliations:** 1 Institute for Global Food Security, School of Biological Sciences, Medical Biology Centre, Queen’s University Belfast, 97 Lisburn Road, Belfast BT9 7BL, UK

## Abstract

**Background:**

In neutralophilic bacteria, monovalent metal cation/H^+^ antiporters play a key role in pH homeostasis. In *Escherichia coli*, only four antiporters (NhaA, NhaB, MdfA and ChaA) are identified to function in maintenance of a stable cytoplasmic pH under conditions of alkaline stress. We hypothesised that the multidrug resistance protein MdtM, a recently characterised homologue of MdfA and a member of the major facilitator superfamily, also functions in alkaline pH homeostasis.

**Results:**

Assays that compared the growth of an *E. coli* Δ*mdtM* deletion mutant transformed with a plasmid encoding wild-type MdtM or the dysfunctional MdtM D22A mutant at different external alkaline pH values (ranging from pH 8.5 to 10) revealed a potential contribution by MdtM to alkaline pH tolerance, but only when millimolar concentrations of sodium or potassium was present in the growth medium. Fluorescence-based activity assays using inverted vesicles generated from transformants of antiporter-deficient (Δ*nhaA*, Δ*nhaB*, Δ*chaA*) *E. coli* TO114 cells defined MdtM as a low-affinity antiporter that catalysed electrogenic exchange of Na^+^, K^+^, Rb^+^ or Li^+^ for H^+^. The K^+^/H^+^ antiport reaction had a pH optimum at 9.0, whereas the Na^+^/H^+^ exchange activity was optimum at pH 9.25. Measurement of internal cellular pH confirmed MdtM as contributing to maintenance of a stable cytoplasmic pH, acid relative to the external pH, under conditions of alkaline stress.

**Conclusions:**

Taken together, the results support a role for MdtM in alkaline pH tolerance. MdtM can therefore be added to the currently limited list of antiporters known to function in pH homeostasis in the model organism *E. coli*.

## Background

The capacity to survive at pH values outside their normal growth range is a prominent feature of many pathogenic bacteria [1]. For example, during their life cycles the neutralophilic enterobacteria *Escherichia coli* and *Vibrio cholerae* can be released into alkaline marine and estuarine environments where they can remain viable and sustain a threat to public health for periods of up to weeks [2,3]. Such alkalitolerance requires neutralophilic bacteria to maintain a stable cytoplasmic pH, in the narrow range of pH 7.4 to 7.8, that is acidic relative to that of the external environment [4]; to achieve this they employ diverse strategies, all specifically designed to contribute to the maintenance of cytoplasmic proton concentration. These include alterations of cell membrane structure and composition to retain protons, remodelling of metabolic patterns to increase acid production, and upregulation of expression and activity of transporters that harvest protons [5]. Active inward transport of protons by cytoplasmic membrane cation/H^+^ antiporters is crucial to the latter strategy and often plays a dominant role in alkaline pH homeostasis in bacteria [6,7].

The transportomes of most free-living bacteria contain numerous integral membrane secondary active cation/H^+^ antiporters that can couple the inward movement of protons to the outward movement of either Na^+^ or K^+^ ions in a process driven by the proton motive force (PMF) [7]. To date, only a few of the transporters likely to be involved in alkaline pH homeostasis by neutralophilic bacteria have been identified and characterised. Nevertheless, studies of specific sodium/proton (Na^+^/H^+^) and potassium/proton (K^+^/H^+^) antiporters have helped illuminate their individual contributions to this process. In *E. coli* alkaline pH homeostasis is realised by the combined and partially overlapping functions of at least three such transporters: the paradigm Na^+^/H^+^ antiporter NhaA [8]; MdfA, a well-characterised Na^+^/(K^+^)/H^+^ antiporter that was first identified as a multidrug-resistance transporter [9] belonging to the ubiquitous, large and diverse major facilitator superfamily (MFS)[10,11]; and the K^+^/(Na^+^)(Ca^2+^) /H^+^ antiporter ChaA [12]. NhaA is dominant in the alkaline pH range of up to pH 9, and it confers alkalitolerance to cells only in the presence of externally added Na^+^[13]. Furthermore, *nhaA* deletion mutants can only grow at alkaline pH in the absence of external Na^+^ ions [14]. MdfA overexpressed from a multicopy plasmid extends the alkalitolerance of *E. coli* cells up to pH 10 when Na^+^ or K^+^ is added to the external growth medium, and MdfA can take over from NhaA when the latter is deleted or dysfunctional [9]. Finally, ChaA is active at pH values above 8.0 in the presence of external K^+^ and it supports alkaline pH homeostasis by coupling the efflux of intracellular K^+^ to the uptake of protons [12]. The role of MdfA in alkaline pH homeostasis is of particular interest considering its contribution to multidrug resistance in *E. coli*[15]. Like MdfA, other multidrug transporters of the MFS are polyspecific with respect to substrate recognition profile, and they can efflux a remarkably diverse range of substrates from bacterial cells [16]. Interest in these proteins is further compounded by the recent shift in perception that they function not merely as part of a defensive response to drugs, but as vital components of other fundamental physiological processes in bacteria [17-20]; despite this, a function independent of multidrug efflux has been described for very few of them [9,21-23]. Working from this perspective, we hypothesised that multidrug efflux proteins other than MdfA could play a role in pH homeostasis in *E. coli*. One candidate is the 12-transmembrane spanning segment drug/H^+^ antiporter MdtM, a recently characterised member of the MFS that contributes to intrinsic resistance of *E. coli* to a broad spectrum of antimicrobials including ethidium bromide (EtBr), chloramphenicol, and several quaternary ammonium compounds [24,25].

Here we show through a combination of cell growth studies, transport assays using whole cells and inverted vesicles, and measurements of intracellular pH, that MdtM is required for adaptation of *E. coli* to alkaline environments and that the observed alkalitolerance is due to a monovalent metal cation/H^+^ antiport activity of MdtM that functions to maintain a cytoplasm that is acidic relative to the outside of the cell; this activity is only apparent at distinct alkaline pH values of between pH 9 and pH 10, and in the presence of Na^+^ or K^+^ ions in the growth medium. As such, MdtM represents a novel and functionally versatile *E. coli* Na^+^(K^+^)/H^+^ antiporter that functions in alkaline pH homeostasis within a defined basic pH range.

## Results

### *E. coli* cells devoid of MdtM are sensitive to alkaline pH

**Figure 1:**
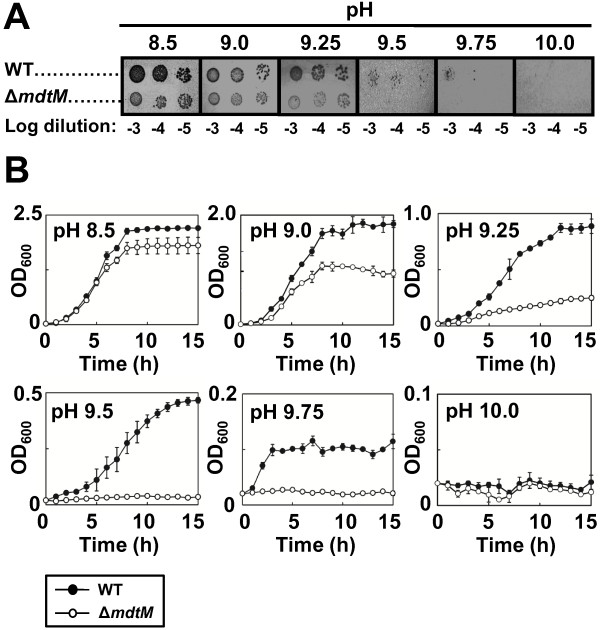
**Effect of chromosomal deletion of *mdtM *on growth of *E. coli *cells at alkaline pH.** (**A**) Growth phenotypes of wild-type (WT) and *mdtM*-deletion mutant (Δ*mdtM*) *E. coli* BW25113 cells grown at different alkaline pH’s on LB agar. As indicated, 4 μl aliquots of a logarithmic dilution series of cells were spotted onto the solid media and the plates were incubated for 24 h at 37°C prior to digital imaging. (**B**) Growth of wild-type and Δ*mdtM E. coli* BW25113 cells in liquid LB media at different alkaline pH values. Data points and error bars represent the mean ± SE of three independent measurements.

To investigate a physiological role for MdtM in basic pH tolerance we characterised the growth of wild-type and Δ*mdtM* single-deletion mutant *E. coli* BW25113 cells under various alkaline pH conditions in both solid and liquid media (Figure 1). On LB-agar plates, both strains exhibited similar growth at pH values of 8.5 to 9.25 (Figure 1A). However, as the pH of the media increased beyond pH 9.25, the growth of Δ*mdtM* cells was inhibited compared to wild-type cells and only the latter exhibited colony formation at pH 9.5 and pH 9.75. No colonies formed at pH 10. The growth assays in liquid media corroborated the results of the solid media assays and highlighted the deleterious effect of the chromosomal *mdtM* deletion on alkalitolerance under the experimental conditions employed (Figure 1B). At pH 8.5, the wild-type cells grew slightly better than those of the single-deletion mutant. However, as the pH of the medium was increased the effect of the *mdtM* deletion became more pronounced; at pH 9.0 and pH 9.25 the wild-type cells grew relatively well whereas the growth of the deletion mutant was suppressed, and even at pH 9.5 and 9.75 the wild-type cells still grew, albeit to a low density. Strikingly, at the latter pH values, growth of the deletion mutant was completely arrested. Neither strain grew at pH 10. Together, these data suggest a role for MdtM in conferral of alkalitolerance to *E. coli* cells within a narrow pH window framed by pH 9 and pH 10.

### *E. coli* cells expressing MdtM from multicopy plasmid display an alkalitolerant phenotype

**Figure 2:**
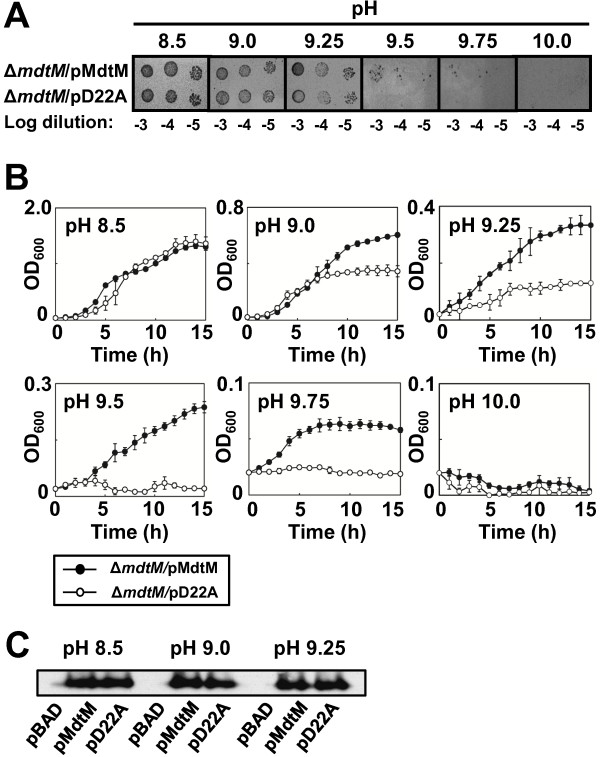
***E. coli *Δ*mdtM *cells complemented with wild-type *mdtM *can grow at alkaline pH.** (**A**) Growth phenotypes of Δ*mdtM E. coli* BW25113 cells transformed with a multicopy plasmid encoding wild-type MdtM (pMdtM) or the dysfunctional MdtM D22A mutant (pD22A) at different alkaline pH’s on LB agar. As indicated, 4 μl aliquots of a logarithmic dilution series of cells were spotted onto the solid media and the plates were incubated for 24 h at 37°C prior to digital imaging. (**B**) Growth of Δ*mdtM E. coli* BW25113 cells complemented with pMdtM or the pD22A mutant in liquid LB media at different alkaline pH values. Data points and error bars represent the mean ± SE of three independent measurements. (**C**) Comparison of expression levels of recombinant wild-type and D22A mutant MdtM at three different pH values by Western blot analysis of DDM detergent-solubilised membranes of *E. coli* BW25113 cells that overproduced the protein from plasmidic DNA. Cells harbouring empty pBAD vector were used as a negative control. Each lane contained 10 μg of membrane protein.

To further test the role of MdtM in alkalitolerance, we assayed growth of Δ*mdtM* cells expressing MdtM from a multicopy plasmid under control of the non-native *araBAD* promoter under basic pH conditions in solid and liquid media. The energetic costs of overexpressing the transporter resulted in differences in the growth characteristics displayed by cells harbouring plasmidic MdtM compared to those harbouring plain vector alone (data not shown). To account for this, Δ*mdtM* cells that overproduced dysfunctional MdtM from the pD22A plasmid were used as a control [24]. As shown in Figure 2A, on solid medium at pH 8.5, cells that overexpressed the dysfunctional transporter grew as well as those that overproduced wild-type MdtM. However, as the pH of the medium became more alkaline, growth of cells that synthesised the D22A mutant was progressively inhibited until, at pH 9.5 and 9.75, only the cells that overproduced functional MdtM were capable of colony formation. Both strains failed to grow on solid medium buffered to pH 10. Again, the results of the assays performed on solid medium were corroborated by assays performed in liquid medium (Figure 2B). The latter confirmed that growth of Δ*mdtM* cells complemented with pD22A was completely arrested above pH 9.25 whereas cells complemented with plasmidic DNA that encoded wild-type MdtM still retained capacity for limited growth up to a pH of at least 9.75. Liquid medium buffered to pH 10 did not support growth of either strain.

To provide further confirmation of the contribution of overproduced MdtM to an alkalitolerant phenotype, and to nullify any contribution by chromosomally-encoded MdfA in the previously described experiments, a set of growth assays that used the Δ*mdfA* BW25113 strain complemented with pMdtM and the pD22A mutant were performed in liquid medium buffered to pH values of between 8.5 and 9.5 (see Additional file 1). As observed in the assays that utilised Δ*mdtM* cells transformed with pMdtM and pD22A, there was no difference in the growth characteristics of Δ*mdfA* transformants cultured at pH 8.5 (see Additional file 1; top left panel). However, as the pH of the growth medium was made more alkaline the Δ*mdfA* pD22A transformants again became increasingly inhibited until, at pH 9.5, their growth was essentially halted (see Additional file 1; bottom right panel). In contrast, Δ*mdfA* cells that overproduced plasmidic, wild-type MdtM grew at all the alkaline pH values tested, thus underlining the ability of overexpressed MdtM to compensate for loss of MdfA and thereby support an alkalitolerant phenotype of *E. coli*.

Finally, to ensure that the observed differences in the cell growth assays were not due simply to differences in the expression levels of the wild-type and D22A mutant transporter, Western blot analysis of dodecyl-β-D-maltopyranoside (DDM) detergent-solubilized cytoplasmic membranes from each strain grown at different pH values was performed (Figure 2C). The analysis confirmed that the wild-type and mutant transporter were not only correctly targeted to the inner membrane but also that each was overexpressed to similar levels irrespective of the pH of the growth medium. Collectively, these results demonstrate that MdtM can confer *E. coli* with tolerance to alkaline pH values up to 9.75, provided it is functionally expressed from a multicopy plasmid.

### Na^+^ or K^+^ cations are required for MdtM-mediated alkaline pH tolerance

**Figure 3:**
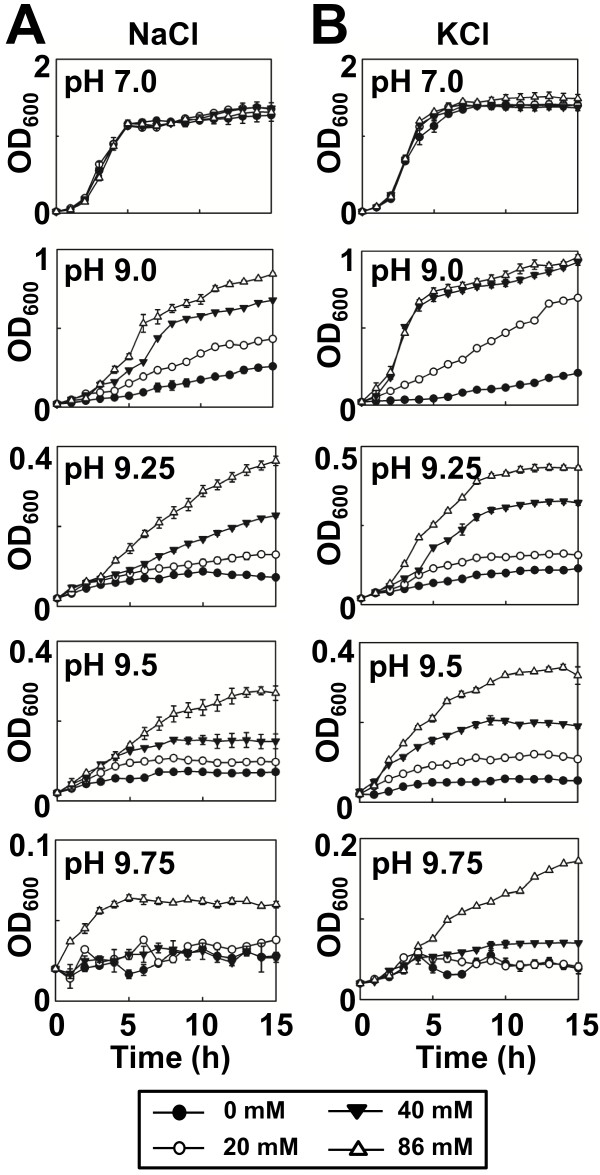
***E. coli *cells complemented with *mdtM *require sodium or potassium for growth at alkaline pH.** Growth of *E. coli* BW25113 Δ*mdt*M cells complemented with plasmidic, wild-type *mdtM* in salt-free liquid medium supplemented with either 0 mM, 20 mM, 40 mM or 86 mM NaCl (**A**) or KCl (**B**). Data points and error bars represent the mean ± SE of three independent experiments.

Inward active transport of protons by antiporters involved in alkaline pH homeostasis in bacteria is usually driven by outward co-transport of monovalent cations such as Na^+^ or K^+^[1]. Therefore, we characterised the requirement of Na^+^ or K^+^ for MdtM-mediated alkalitolerance by performing growth experiments with *E. coli* BW25113 Δ*mdt*M cells complemented with pMdtM in salt-free liquid medium supplemented with different concentrations (ranging from 20 mM to 86 mM) of NaCl or KCl at different pH values. Cells grown at neutral pH did not exhibit any Na^+^ or K^+^-dependence (Figure 3A and B, top panels). However, as pH of the medium increased, cell growth showed distinct NaCl or KCl concentration dependence, suggesting that the presence of Na^+^ or K^+^ ions is required for MdtM-mediated basic pH tolerance (Figure 3). Notably, at alkaline pH, cells grown in the presence of the higher concentrations of K^+^ (Figure 3B) achieved higher optical density than those grown in the presence of the corresponding concentrations of Na^+^ (Figure 3A). The stronger growth of cells observed in the presence of K^+^ in the external medium probably reflects the activity of the chromosomally encoded ChaA K^+^/H^+^ antiporter [12].

**Figure 4:**
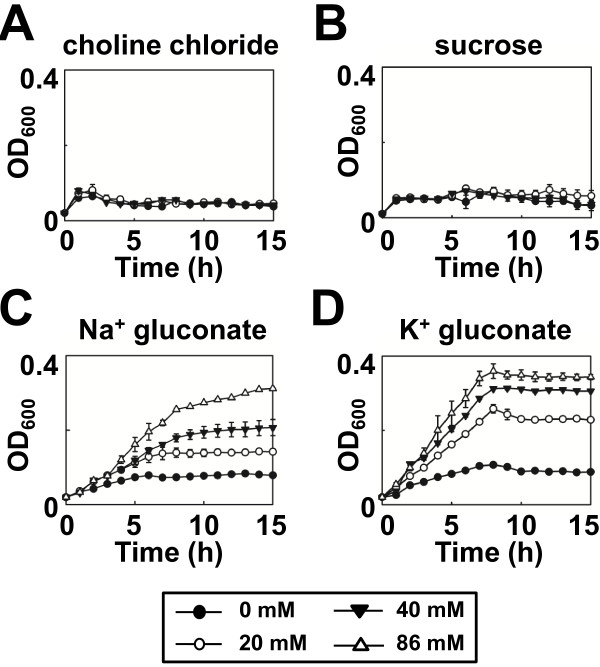
**Choline, chloride or sucrose do not support growth of *E. coli *cells complemented with *mdtM *at alkaline pH.** Growth of *E. coli* BW25113 Δ*mdt*M cells complemented with wild-type *mdtM* in salt-free liquid medium buffered to pH 9.5 in the presence of 0 mM, 20 mM, 40 mM or 86 mM choline chloride (**A**), sucrose (**B**), sodium gluconate (**C**) and potassium gluconate (**D**). Data points and error bars represent the mean ± SE of three independent experiments.

Cells were unable to grow in liquid medium in which choline chloride (Figure 4A) or sucrose (Figure 4B) replaced the chloride salt of sodium or potassium, thereby negating a role for either chloride ions or osmotic pressure in MdtM-mediated alkalitolerance. Further evidence of a dependence upon Na^+^ or K^+^, but not Cl^-^, for alkalitolerance came from growth experiments performed in medium containing either sodium gluconate (Figure 4C) or potassium gluconate (Figure 4D); both these compounds supported the growth of MdtM-expressing cells at pH 9.5 and did so in a concentration-dependent manner that reflected the results of the growth experiments performed in liquid medium containing NaCl or KCl (Figure 3). As observed in the experiments that tested the effects of added NaCl and KCl on cell growth at alkaline pH values, cells grown at pH 9.5 in the presence of added K^+^ gluconate achieved higher optical densities at all the concentrations tested than those cultured in medium that contained Na^+^ gluconate.

**Figure 5:**
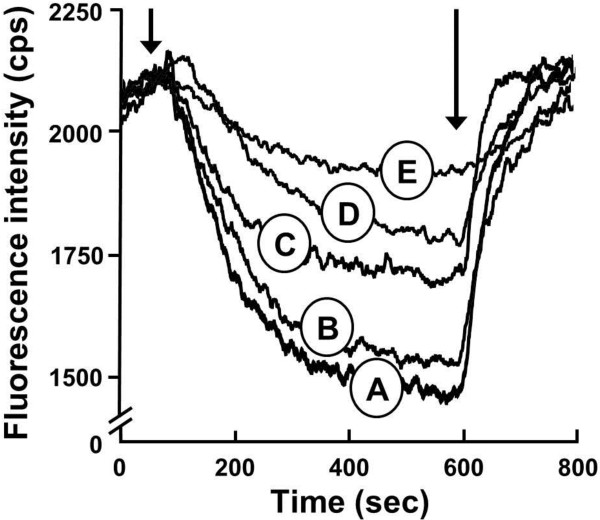
**Whole cell ethidium bromide transport assays performed in the presence of different concentrations of NaCl.** Representative traces of the efflux of EtBr from cells expressing wild-type MdtM in the presence of 0 mM (**A**), 20 mM (**B**), 50 mM (**C**) and 100 mM (**D**) NaCl. EtBr efflux was monitored continuously by measuring fluorescence emission at 600 nm upon excitation at 545 nm. UTL2 cells that expressed the MdtM D22A mutant in the absence of added NaCl were used as a control (**E**). Cells loaded with EtBr were energised by addition of glucose (as indicated by the first arrow) and efflux of EtBr was monitored for 800 s. CCCP (100 μM) was added (as indicated by the second arrow) to abolish active transport. Fluorescence intensity was measured in counts per second (cps).

A further indication that the observed alkalitolerance was mediated by MdtM-catalysed monovalent metal cation transport came whole cell transport assays that used fluorescence spectroscopy measurements of the effects of increasing concentrations of NaCl on the EtBr efflux activity of pMdtM transformants of *E. coli* UTL2 cells (Figure 5). In the absence of NaCl, addition of 0.5% (w/v) glucose to energize the cells resulted in a steady decrease in the fluorescence intensity as EtBr was actively extruded against its concentration gradient (Figure 5, trace A). Dissipation of the proton electrochemical gradient by addition of the ionophore carbonyl cyanide 3-chlorophenylhydrazone (CCCP) caused the fluorescence signal to rise again, indicating disruption of EtBr efflux. In contrast to the results obtained from MdtM-expressing cells, the fluorescence of control cells that expressed the dysfunctional MdtM D22A mutant decreased more slowly and by a much smaller amount over the timescale of the assay (Figure 5, trace E). In this control the residual EtBr efflux is likely due to the activity of chromosomally encoded transporters that recognise EtBr as a substrate. As expected, the addition of 100 mM NaCl to control cells harbouring pD22A had no noticeable effect on the shape or magnitude of the trace (data not shown). In contrast, addition of Na^+^ cations to UTL2 cells transformed with pMdtM clearly inhibited EtBr efflux (Figure 5, traces B, C and D). Moreover, this inhibition was titratable; addition of increasing concentrations of Na^+^ resulted in an increasing inhibition of EtBr efflux. Addition of choline chloride had no measurable effect on EtBr efflux (data not shown), thereby establishing that the inhibition of EtBr efflux by NaCl was due solely to Na^+^ ions. Together, the results of the whole cell transport assays suggest that EtBr and Na^+^ utilise the same binding site and/or translocation pathway in MdtM. Indeed, in the closely related MdtM homolog MdfA, the multidrug and Na^+^ cation translocation pathways overlap [9].

### MdtM catalyses K^+^/H^+^ and Na^+^/H^+^ exchange activities

**Figure 6:**
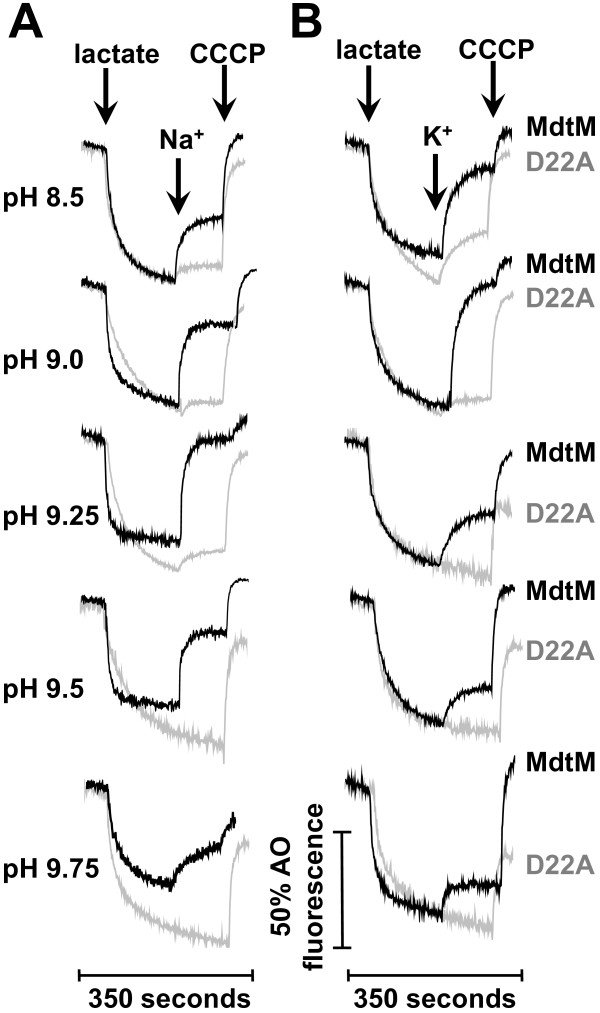
**Cation-driven proton translocation by MdtM.** Cation-driven proton translocation by MdtM at alkaline pH was measured by the fluorescence dequenching of acridine orange upon addition of Na^+^ gluconate (**A**) or K^+^ gluconate (**B**) to inverted vesicles derived from antiporter-deficient *E. coli* TO114 cells that overexpressed recombinant wild-type MdtM (black traces) or the dysfunctional MdtM D22A mutant (grey traces). Respiration-dependent generation of ΔpH (acid inside) was established by addition of lactate as indicated and once the fluorescence quench of acridine orange reached a steady state, Na^+^ gluconate or K^+^ gluconate was added to a final concentration of 100 mM. Addition of 100 μM CCCP at the time indicated was used to completely dissipate ΔpH. The traces are representative of experiments performed in triplicate on at least two separate preparations of inverted vesicles. The fluorescence intensity of each measurement is represented as a percentage of the initial acridine orange fluorescence signal prior to addition of lactate.

The growth assay and whole cell EtBr efflux data implied that MdtM-catalysed K^+^/H^+^ and Na^+^/H^+^ antiport activities underpinned alkalitolerance. To examine if MdtM mediated the exchange of K^+^ and Na^+^ for protons, we measured the changes in luminal pH of inverted membrane vesicles generated from antiporter-deficient TO114 cells [26] that overexpressed wild-type MdtM by monitoring the fluorescence dequenching of acridine orange upon addition of Na^+^ gluconate or K^+^ gluconate to the transport assay buffer at the indicated alkaline pH values (Figure 6). Inverted vesicles prepared from TO114 cells that overproduced dysfunctional MdtM D22A mutant were used as controls.

The control vesicles (Figure 6; grey traces) exhibited negligible Na^+^/H^+^ or K^+^/H^+^ activities at pH values of 9.0 to 9.75. This was expected because the TO114 cells from which the inverted vesicles were generated are devoid of the major antiporters NhaA, NhaB and ChaA that function primarily in monovalent metal cation/H^+^ exchange at alkaline pH [12,26]. However, at pH 8.5 the controls exhibited some degree of exchange activity; this activity was more pronounced upon addition of K^+^ ions and resulted in ~30% dequenching of the initial lactate-induced fluorescence quench (Figure 6B, top panel). It is conceivable that this dequenching was due to the activity of other, chromosomally-encoded antiporters that operate in the same pH range and that have a greater affinity for K^+^ than Na^+^ ions. In all control experiments, addition of 100 μM CCCP at the time indicated resulted in dissipation of the ΔpH, as revealed by an instantaneous dequenching of the fluorescence signal. This confirmed that the inverted vesicles had maintained integrity over the lifetime of the assay.

In contrast to the controls, addition of Na^+^ or K^+^ to inverted vesicles containing recombinant wild-type MdtM resulted in a rapid and significant dequenching of the lactate-induced, acridine orange steady state fluorescence at all the alkaline pH values tested (Figure 6; black traces), thus indicating that MdtM was responsible for catalysing both Na^+^/H^+^ and K^+^/H^+^ exchange reactions. The magnitude of the dequenching at each pH value, however, varied depending upon the pH and the metal cation added; in the case of added Na^+^ the most pronounced dequenching was observed at pH 9.25 (Figure 6A; black traces) whereas the maximal K^+^-induced dequenching occurred at pH 9.0 (Figure 6B; black traces). As observed from the assays performed on control vesicles, the addition of CCCP to the reaction mixtures resulted in a further dequenching of the fluorescence signal, confirming that the MdtM-containing inverted vesicles had also maintained integrity for the lifetime of the assay.

### pH profiles of MdtM-catalysed K^+^/H^+^ and Na^+^/H^+^ exchange activities

**Figure 7:**
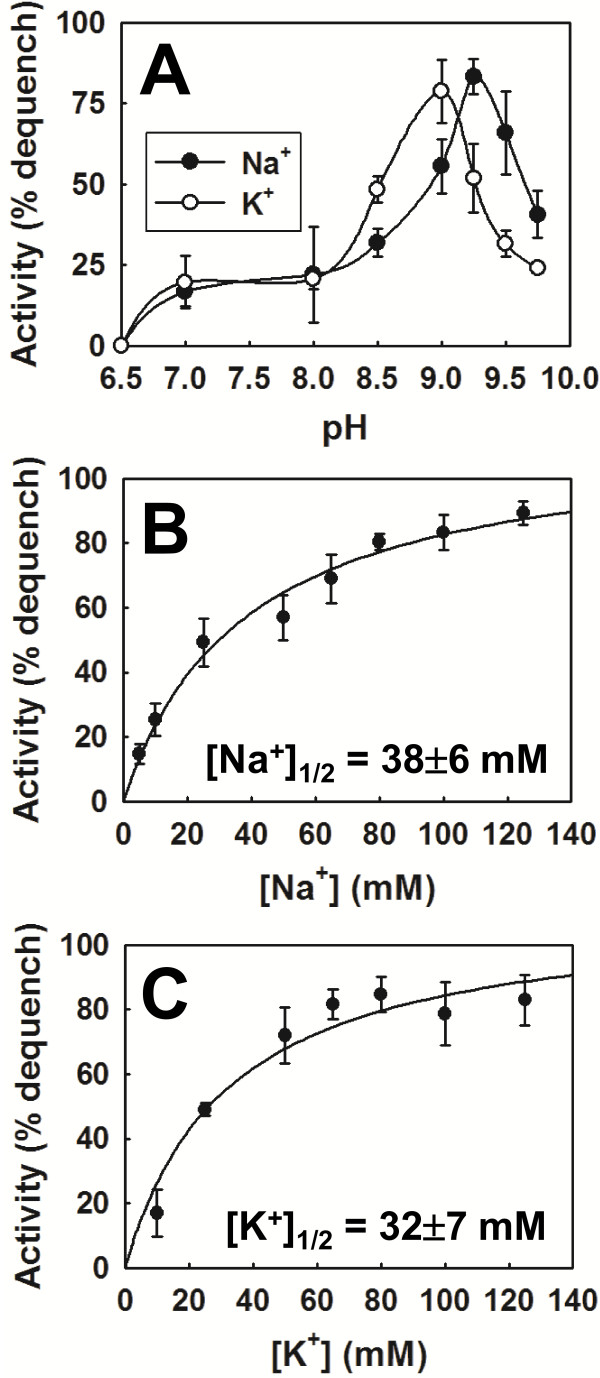
**The pH profile and apparent affinity of MdtM for Na**^**+ **^**and K**^**+**^**.** (**A**) The pH profile of MdtM-mediated Na^+^/H^+^ and K^+^/H^+^ antiport activity. Transporter activity at each pH value was calculated as described in Methods. (**B**) The concentration of Na^+^ and (**C**) K^+^ required for the half-maximal acridine orange fluorescence dequenching response was estimated from measurements of the antiport activity of wild-type recombinant MdtM as a function of cation concentration at the previously determined pH optimum for each antiport reaction (pH 9.25 for Na^+^/H^+^ exchange and pH 9.0 for K^+^/H^+^ exchange). The [Na^+^]_1/2_ and [K^+^]_1/2_ values are an indication of the affinity of MdtM for each cation. In each panel, the data represent the mean ± SD of three independent experiments.

Measurements of the acridine orange fluorescence dequenching enabled a plot of the K^+^/H^+^ and Na^+^/H^+^ exchange activities (expressed as the percentage dequenching of the lactate-induced fluorescence quenching) as a function of pH to be constructed, and this revealed a clear pH-dependence for both (Figure 7A). At pH ≤6.5, no transport of the probed K^+^ and Na^+^ cations was detected, providing further evidence that MdtM does not operate as a monovalent metal cation/H^+^ antiporter at acidic pH. However, as the pH increased and became more alkaline, a significant exchange activity was recorded. From no detectable activity at pH 6.5, the activity of MdtM in the presence of 100 mM Na^+^ or K^+^ increased to ~20% dequenching at the pH range of 7.0 to 8.0 (Figure 7A). Between pH 8.0 and 9.75, the pH profiles for both exchange activities were essentially bell-shaped, with the activity optimum for MdtM-catalysed K^+^/H^+^ antiport at pH 9.0 and that of Na^+^/H^+^ antiport at pH 9.25. The activity of MdtM at each pH optimum was similar, attaining a mean corrected fluorescence dequenching of ~ 80%.

### Apparent affinity of MdtM for transported Na^+^ and K^+^ is low

To permit a crude assessment of the affinity of MdtM for the transported metal cations, a series of dose–response experiments, covering substrate ranges of 5 mM - 125 mM Na^+^ and K^+^ (Figures 7B & C), were performed on inverted vesicles at the pH optimum of each substrate using the acridine orange fluorescence quenching /dequenching assay as described in the Methods section. Although it was not possible to access actual *K*_m_ values using these assays, they did permit the concentrations of Na^+^ and K^+^ required for the half-maximal response to be estimated and the results implied that MdtM has low apparent affinity for monovalent metal cations, with [Na^+^]_1/2_ of 38±6 mM (Figure 7B) and [K^+^]_1/2_ of 32±7 mM (Figure 7C).

### MdtM also catalyses Rb^+^/H^+^ and Li^+^/H^+^ antiport but not Ca^2+^/H^+^ exchange

**Figure 8:**
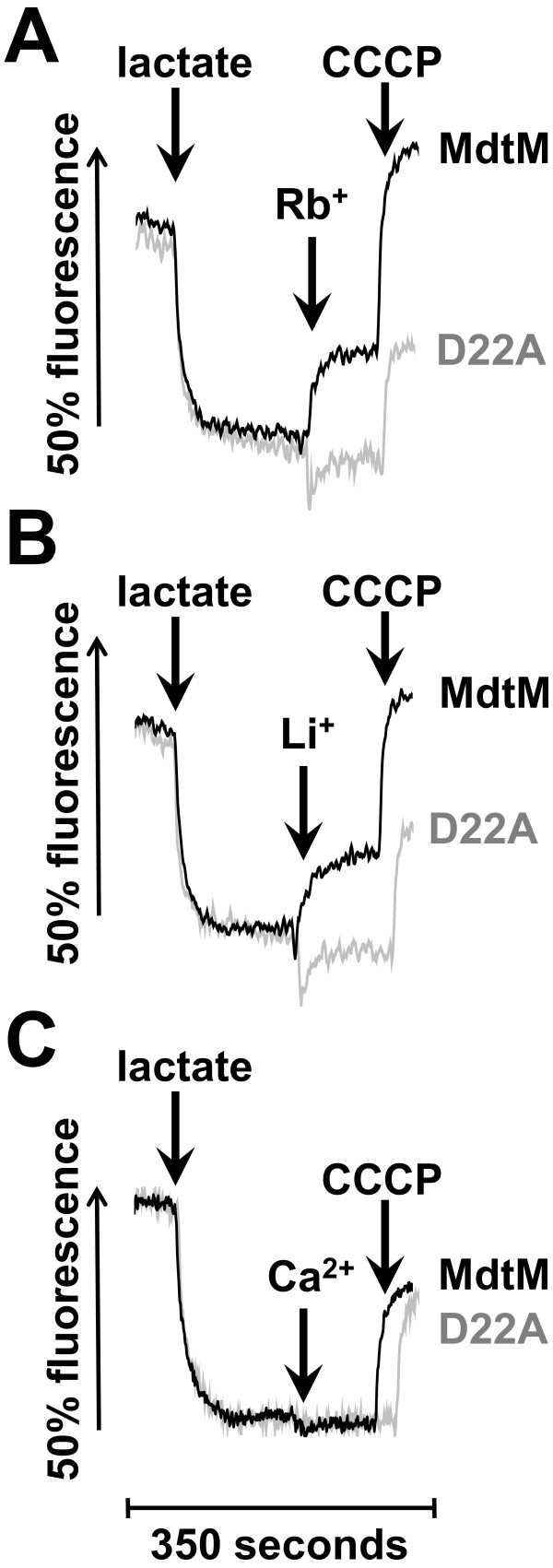
**MdtM-catalysed Rb**^**+**^**/H**^**+**^**, Li**^**+**^**/H**^**+ **^**and Ca**^**2+**^**/H**^**+ **^**exchange at alkaline pH.** Exchange was determined by the fluorescence dequenching of acridine orange in inverted vesicles derived from antiporter-deficient *E. coli* TO114 cells that overexpressed recombinant wild-type MdtM (black traces) or the dysfunctional MdtM D22A mutant (grey traces). A ΔpH across the vesicle membrane was established by addition of lactate as indicated and once the fluorescence quench of acridine orange achieved a steady state, 40 mM Rb_2_SO_4_ (**A**), 40 mM Li_2_SO_4_ (**B**) or 40 mM CaSO_4_ (**C**) was added to the vesicles. Addition of 100 μM CCCP abolished the ΔpH. The fluorescence intensity of each measurement is represented as a percentage of the initial acridine orange fluorescence signal prior to addition of lactate. The fluorescence measurements were conducted at pH 9.0 and the traces shown are representative of experiments performed in triplicate on at least two separate preparations of inverted vesicles.

Bacterial Na^+^/H^+^ and K^+^/H^+^ antiporters that function in alkaline pH homeostasis can often also transport cations of other metals such as rubidium, lithium and calcium [12,27-29]. Therefore, the capacity of inverted vesicles of TO114 cells transformed with pMdtM to support the exchange of Rb^+^, Li^+^ and Ca^2+^ for protons was examined at pH 9.0 using the acridine orange fluorescence quenching/dequenching assay. Not unexpectedly, the addition of 40 mM Rb_2_SO_4_ to the inverted vesicles containing wild-type MdtM resulted in ~35% dequenching of the lactate-induced fluorescence quench, indicating that MdtM was capable of catalysing the exchange of the potassium analogue Rb^+^ for protons (Figure 8A; black trace). A similar magnitude of dequenching was observed when 40 mM Li_2_SO_4_ was added to inverted vesicles (Figure 8B; black trace), confirming that Li^+^/H^+^ exchange is also catalysed by MdtM. In contrast, the addition of 40 mM CaSO_4_ to inverted vesicles did not elicit a dequenching of the respiration induced fluorescence quenching (Figure 8C; black trace), demonstrating that Ca^2+^ is not a substrate for MdtM under the conditions tested; this assay also confirmed that the activity observed upon addition of the sulphate salts of Rb^+^ and Li^+^ to the inverted vesicles was not due to any MdtM-mediated exchange of sulphate anions for protons. Additionally, magnesium sulphate or choline chloride at final concentrations of 40 mM also failed to dequench the fluorescence (data not shown). Control assays conducted with inverted vesicles that contained the dysfunctional MdtM D22A mutant did not exhibit any fluorescence dequenching in response to the addition of any of the cations tested (Figure 8; grey traces), thereby providing further robust evidence that the dequenching observed upon the addition of Rb^+^ and Li^+^ to vesicles generated from TO114 cells transformed with pMdtM was due to a process mediated by the functionally expressed recombinant transporter.

### MdtM-catalysed K^+^/H^+^ and Na^+^/H^+^ antiport is electrogenic

**Figure 9:**
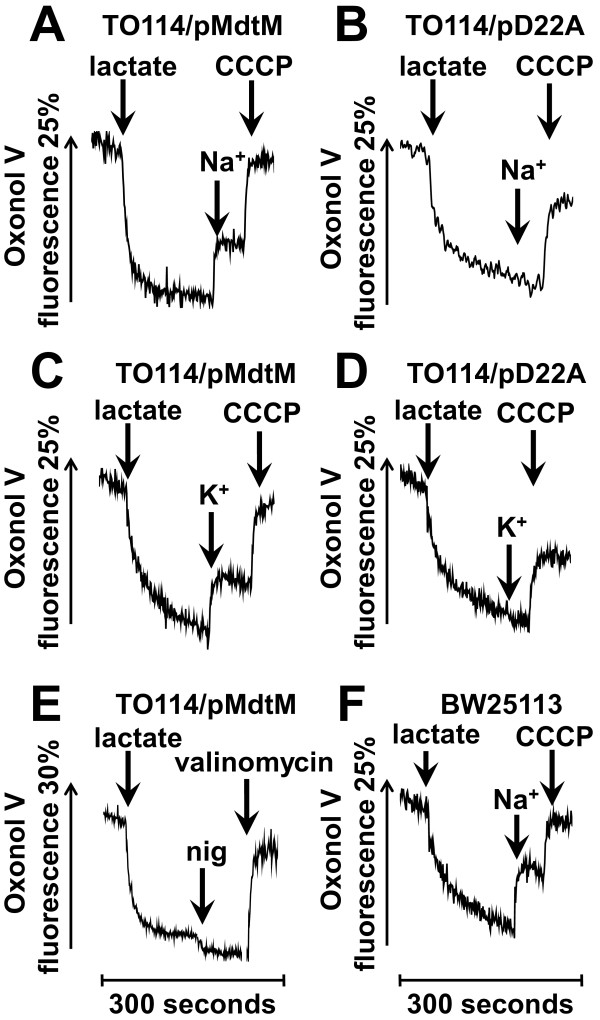
**The electrogenicity of MdtM-catalysed Na**^**+**^**/H**^**+ **^**and K**^**+**^**/H**^**+ **^**antiport.** The electrogenicity of MdtM-catalysed Na^+^/H^+^ and K^+^/H^+^ antiport at alkaline pH was probed by Oxonol V fluorometry of inverted vesicles generated from *E. coli* TO114 cells transformed with pMdtM (**A, C & E**) or, as a negative control, pD22A (**B & D**). Inverted vesicles isolated from BW25113 cells were used as a positive control (**F**). Respiration-dependent formation of Δψ was initiated by addition of lactate at the time indicated. Once steady-state Δψ was achieved, antiport was initiated by addition of 100 mM Na^+^ gluconate (**A & B**) or 100 mM K^+^ gluconate (**C & D**) as indicated. Vesicles were depolarised by addition of CCCP or valinomycin in the presence of K^+^ as indicated. Fluorescence measurements on TO114 inverted vesicles were conducted at either pH 9.0 (for detection of K^+^/H^+^ antiport; **panels C & D**) or pH 9.25 (for detection of Na^+^/H^+^ antiport; **panels A & B**), whereas positive control measurements using vesicles derived from BW25113 cells were done at pH 8.5 to ensure detection of the activity of the electrogenic antiporter, NhaA (**panel F**). The Oxonol V fluorescence is presented as a percentage of the initial fluorescence prior to establishment of the steady-state Δψ. The traces shown are representative of experiments performed in triplicate on two separate preparations of inverted vesicles.

Generally, cation/proton antiporters involved in alkaline pH homeostasis are required to mediate an electrogenic antiport that is energized by the transmembrane electrical potential, Δψ [5]. Therefore, to probe whether MdtM catalyses electrogenic antiport, inverted vesicles were generated from TO114 cells transformed with pMdtM and assayed for electrogenicity in a chloride-free and potassium-free buffer using the Δψ–sensitive fluorophore Oxonol V. Inverted vesicles produced from TO114 cells transformed with pD22A were used as a negative control. In all the assays, energization of the vesicles by lactate resulted in a rapid quench of Oxonol V fluorescence indicating the generation of respiratory Δψ (Figure 9). To ensure the suitability of the experimental conditions for detection of electrogenic antiport, a positive control (Figure 9F) was performed using inverted vesicles produced from *E. coli* BW25113 cells that contained a full complement of electrogenic antiporters. This control experiment was performed at pH 8.5 to specifically enable detection of NhaA-catalysed, electrogenic Na^+^/H^+^ exchange [30]. Addition of Na^+^ to these vesicles caused a rapid partial dequenching of the Oxonol V fluorescence, indicating electrogenic antiport. Addition of the protonophore CCCP at the time indicated resulted in dissipation of the respiratory Δψ.

Addition of Na^+^ (Figure 9A) or K^+^ (Figure 9C) to inverted vesicles produced from TO114 cells that overexpressed wild-type recombinant MdtM resulted in a partial depolarization of Δψ, whereas addition of the same metal cations to negative control vesicles containing dysfunctional MdtM resulted in no detectable depolarization (Figures 9B and 9D). In each case, addition of the protonophore CCCP at the times indicated resulted in dissipation of Δψ. In another control experiment, addition of the ionophore nigericin to TO114/pMdtM vesicles pre-incubated in the presence of 50 mM K^+^ gluconate resulted in a small increase in the magnitude of Δψ due to conversion of ΔpH to Δψ by the electroneutral K^+^/H^+^ exchange activity of nigericin (Figure 9E). Addition of valinomycin to the same vesicles at the time indicated completely dissipated Δψ.

Together, these qualitative data clearly indicate that MdtM-catalysed Na^+^/H^+^ and K^+^/H^+^ antiport at alkaline pH is electrogenic, with > 1 H^+^ exchanged per Na^+^ or K^+^; quantitative determination of the stoichiometry of antiport will require reconstitution of purified MdtM into proteoliposomes as was done for NhaA [31], and this is the subject of ongoing work by our laboratory.

### In alkaline environments, MdtM functions to maintain a cytoplasmic pH that is acidic relative to external pH

**Figure 10:**
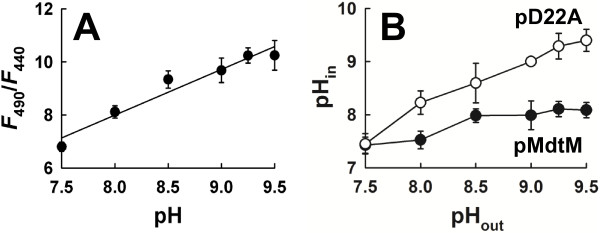
**Measurements of cytoplasmic pH.** (**A**) Calibration plot that correlates the 490 nm/440 nm fluorescence emission ratio of BCECF-AM upon excitation at 530 nm to pH. (**B**) Intracellular pH of *E. coli* BW25113 Δ*mdtM* cells transformed with pMdtM or pD22A as a function of external alkaline pH. In both (**A**) and (**B**) the data points and error bars represent the mean ± SD of three independent measurements.

Taken together, all the previous data strongly support the idea that MdtM contributes to cytoplasmic pH homeostasis under conditions of alkaline stress. Therefore, to demonstrate directly a role for MdtM in this process, *in vivo* measurements of the intracellular pH of *E. coli* BW25113 Δ*mdtM* transformed with pMdtM or pD22A at different external alkaline pH values between pH 7.5 and pH 9.5 were performed in the presence of NaCl using fluorescence measurements of the free acid of the pH-sensitive probe 2,7-bis-(2-carboxyethyl)-5-(and-6)-carboxyfluorescein acetoxymethyl ester (BCECF-AM). Calibration of our system resulted in a reasonably linear correlation between intracellular pH and the 490 nm/440 nm fluorescence ratio over a range of pH values from 7.5 to 9.5 (Figure 10A) thereby making internal cellular pH measurements over this range amenable. The intracellular pH of cells that overexpressed wild-type MdtM from a multicopy plasmid remained relatively constant (at between pH 7.5 and 8.0) over the range of external alkaline pH values tested (Figure 10B; filled symbols). In contrast, cells expressing the dysfunctional D22A mutant of the transporter were unable to maintain a stable cytoplasmic pH, acidic relative to the outside; as the external pH increased there was a concomitant alkalinisation of the cell cytoplasm (Figure 10B; empty symbols). These results uphold our contention that MdtM contributes to alkaline pH homeostasis in *E. coli*.

## Discussion

The chief strategy employed by *E. coli* to maintain a stable cytoplasmic pH under conditions of alkaline challenge is that of proton uptake mediated by cytoplasmic membrane cation/H^+^ antiporters [1]. Until now, only four of this type of antiporter were identified unambiguously to function in alkaline pH homeostasis in *E. coli*; NhaA [32], NhaB [27], MdfA [9] and ChaA [12], and each has different value to the cell depending on the external environmental conditions [1,5,6]. The data presented here define another integral membrane protein, MdtM, a promiscuous multidrug resistance protein belonging to the MFS of secondary active transporters [24], as contributing to alkalitolerance in *E. coli*. MdtM comes into play at a distinct pH range of between 9 and 10 and provides *E. coli* with a sensitive mechanism by which to accommodate proton capture under conditions of alkaline stress.

Analysis of the growth phenotype of the *E. coli* Δ*mdtM* deletion mutant revealed a potential physiological role for MdtM in pH homeostasis (Figures 1 and 2). Under the experimental conditions employed, MdtM was required for the growth of *E. coli* in rich medium at alkaline pH values of >9.0 and <10.0, but only when tens of millimolar concentrations of sodium or potassium ions were present in the growth medium (Figure 3). Similar monovalent metal cation-dependent, alkalitolerance functions have been described for the *Bacillus subtilis* tetracycline efflux protein TetL [33] and the *E. coli* multidrug transporter MdfA [9]. MdfA – the best-characterised multidrug transporter of the MFS [34] - is a close homologue of MdtM (41% sequence identity and 62% similarity)[35] that is reported to play a major role in conferral of alkalitolerance in *E. coli* at pH >9.0, and when potassium is the main monovalent metal cation; conditions under which the major Na^+^/H^+^ antiporter NhaA does not operate [9].

The conditions of external pH and cation composition under which MdtM operates are very similar to, and apparently overlap to an extent with those favoured by MdfA. Despite this apparent overlap in functionality, studies by Lewinson et al. [9] that investigated the effect of chromosomal deletion of *mdfA* on growth of the *E. coli* UT5600 strain showed that cells devoid of MdfA could not grow at alkaline pH values > 9.0. This seemingly indicates that chromosomal *mdtM* cannot substitute for *mdfA* to support growth at alkaline pH. Concomitantly, in our growth experiments (Figures 1 and 2), the chromosomal *mdfA* gene was ostensibly incapable of supporting growth of the BW25113 Δ*mdtM* strain at the alkaline pH values tested. These observations raise the question as to why *mdfA* and *mdtM* did not compensate for one another at alkaline pH. This can be rationalised if one considers the multidrug efflux capabilities of these particular transporters; under the experimental conditions employed by our study the BW25113 Δ*mdtM* strain was grown in the presence of the antibiotic kanamycin, a known substrate of MdfA [15], and at the concentration (~60 μM) of kanamycin used for selection of the Δ*mdtM* strain the copies of chromosomally-encoded MdfA may be saturated by the antibiotic and incapable of mediating the low-affinity Na^+^(K^+^)/H^+^ exchange necessary for the protein to contribute to pH homeostasis. Indeed, in a previous study that also used *E. coli* strains that required kanamycin for selection [9], this may be why MdfA was required to be overproduced from a multicopy plasmid to demonstrate its role in pH homeostasis at alkaline pH values >9.0. If MdtM also recognises kanamycin as a substrate, this could account for why copies of chromosomally-encoded MdtM were unable to compensate for the deletion of *mdfA* in the cell growth assays described in [9]. Alternatively, the apparent lack of substitution by the transporters could be explained simply by differences between the bacterial strains used, and/or the experimental conditions employed by each study. If the latter is true, it suggests that each transporter is beneficial to the cell under subtly different environmental conditions. Whatever the explanation, our results remain consistent with a role for MdtM in alkaline pH homeostasis in *E. coli*.

In our growth experiments, the requirement for sodium or potassium ions for MdtM-mediated alkalitolerance suggests a mechanistic role for Na^+^ and K^+^ ions in MdtM activity and this was confirmed by fluorescence-based activity assays performed at alkaline pH values (Figure 6). These assays showed that MdtM catalysed a Na^+^(K^+^)/H^+^ antiport that, in vivo, probably enables the exchange of internal monovalent metal cations for extracellular protons to maintain a stable internal pH, acid relative to outside, during exposure to alkaline environments. This conclusion was supported by our experiments that used BCECF fluorometry to measure cytoplasmic pH under different external alkaline pH conditions (Figure 10).

The ability of MdtM to exchange either Na^+^ or K^+^ cations for protons endows *E. coli* with the flexibility to respond effectively to changes in chemical composition of the environment at alkaline pH. When sodium is available, the Na^+^/H^+^ antiport activity of MdtM can permit growth. Under sodium-poor conditions, or when other Na^+^/H^+^ antiporters are disrupted, regulation of cytoplasmic pH by K^+^/H^+^ antiport activity of MdtM can contribute to alkaline pH homeostasis. Although the contribution of K^+^ concentration to pH homeostasis in *E. coli* is still unclear [6,36], the K^+^/H^+^ antiport activity of MdtM may offer a mechanism for regulating cytoplasmic pH by utilising the outwardly-directed K^+^ gradient to drive proton capture during growth at alkaline pH [5,37]. Provided the rate of MdtM is slower than that of the systems that generate the PMF, and of the uptake systems that bring K^+^ into the cell, MdtM will not act as an uncoupler to dissipate the PMF. Furthermore, in alkaline environments, the same K^+^/H^+^ antiport activity of MdtM has the potential to protect *E. coli* from the toxic effects of high intracellular concentrations of K^+^ and, therefore, to function also in K^+^ homeostasis. Just such a function was identified previously for the *E. coli* ChaA antiporter [12]. Additionally, and in contrast to MdfA, MdtM is capable of transporting lithium ions at alkaline pH (Figure 8B) and it may function physiologically in alkaline pH homeostasis when Li^+^ is present. This highlights further the subtle differences in function that exist between the closely-related MdfA and MdtM transporters, and that lessons learned from one cannot simply be imposed upon the other.

As control of internal pH is, by definition, control of cytoplasmic proton concentration, the requirements of bacterial pH homeostasis dictate the relative magnitudes of the transmembrane proton gradient (ΔpH) and transmembrane electrical potential (Δψ), the two individual components that constitute the PMF. Under alkaline pH stress conditions, maintenance of a stable intracellular pH requires that a net cytoplasmic accumulation of protons must occur; therefore, the ΔpH of neutralophiles like *E. coli* is reversed from the usual orientation of alkaline inside [5] and cannot apparently be used to drive proton uptake into the cell. This is a particular problem when Na^+^/H^+^ antiporters are used for alkaline pH homeostasis because, due to the cytotoxicity of Na^+^[5] it is excluded from the cell and, unlike K^+^, cannot provide an outwardly-directed driving force to support an electroneutral exchange. To overcome this, antiporters such as *E. coli* NhaA [31] and *B. subtilis* TetL [38], utilise Δψ to catalyse electrogenic Na^+^/H^+^ exchange and drive net accumulation of H^+^ to acidify the cytoplasm at alkaline pH in the presence of Na^+^. Intriguingly, the MdtM homologue MdfA can catalyse both electrogenic and electroneutral transport of drug substrates [39]; however, the component of the PMF that MdfA utilises to drive Na^+^/H^+^ or K^+^/H^+^ antiport at alkaline pH has not been reported, although it too is likely to be the Δψ. The results of our fluorescence experiments using the Δψ–sensitive probe Oxonol V revealed that MdtM can utilise Δψ as the driving force at alkaline pH to catalyse an electrogenic Na^+^(K^+^)/H^+^ antiport, i.e., an exchange stoichiometry of >1 H^+^ per monovalent metal cation (Figure 9).

Further evidence to support a physiological role for MdtM in alkaline pH homeostasis was gleaned from estimation of the concentrations of Na^+^ and K^+^ required to elicit the half-maximal fluorescence dequench of acridine orange in inverted vesicles (Figure 7). Other transporters that function in bacterial pH homeostasis, such as *E. coli* NhaB [40], ChaA [12] and MdfA [9], and a sodium-specific Na^+^/H^+^ antiporter from *Vibrio parahaemolyticus*[41], all possess affinity for their respective metal ion substrate(s) in the general millimolar range. Our values of [Na^+^]_1/2_ and [K^+^]_1/2_ of 38±6 mM and 32±7 mM, respectively, although not directly related to actual *K*_m_ values [42], suggest MdtM also possesses relatively low affinity for its cognate metal cations and are therefore consistent with a contributory role for the Na^+^/H^+^ and K^+^/H^+^ antiporter activities of MdtM in alkaline pH homeostasis.

In order to function effectively in pH homeostasis, antiporters must be equipped with sensors of the external and/or cytoplasmic pH that can transduce the changes in pH into changes in transporter activity [5]. The pH profile of MdtM activity (Figure 7A) suggests that, like other antiporters involved in pH homeostasis, it too is capable of sensing and responding to changes in ionic composition, and provides additional support for our contention that the different antiport functions performed by MdtM are dictated by subtle changes in pH and the type of cation present in the external environment. In our experiments, because MdtM expression from a multicopy plasmid was placed under control of a non-native arabinose-inducible promoter, this suggests an ability to sense pH at the protein level. This could be achieved simply by changes in the protonation state(s) of amino acid side chains in the protein. Topology prediction studies [24] of MdtM indicated several ionisable residues, located on the periplasmic and cytoplasmic surfaces of the protein as well as in the putative translocation pore, that could conceivably play a role in pH sensing.

Use of the MdtM D22A mutant as a control in transport assays with inverted vesicles precluded the necessity to reconstitute the transporter into proteoliposomes to study its role in pH homeostasis. The observation that the D22A mutant was dysfunctional in all our assays also sheds more light on the mechanistic role of D22 in MdtM function. Previous work showed that even though the mutant protein could bind either cationic or neutral antimicrobial substrates, it could not translocate them across the membrane [24,25]. It was postulated therefore that the negatively charged side chain of D22 probably functions in proton recognition and may form part of a proton relay network in MdtM [24]. Several other acidic residues (D30, D244, D277 and E280) are embedded in putative membrane-spanning regions of MdtM [24], and these too could potentially contribute to formation of the proton relay. Disruption of this network of negatively-charged residues could be sufficient to abrogate the cation/H^+^ antiport activity of the transporter. Although more investigation is clearly required to dissect the role(s) of acidic residues in MdtM-catalysed antiport, recent work by Fluman et al. [43] proposed that the carboxylic groups of the MdfA E26 (the residue homologous to MdtM D22) and D34 residues are important for proton transport and/or antiport coupling. It is conceivable therefore that MdtM could employ a mechanistic strategy in which H^+^ binding to D22 is a prerequisite for (i) the transport of Na^+^ or K^+^ to support its role in alkaline pH homeostasis; and (ii) the transport of drug substrates to support its role in multidrug resistance. A linkage between alkalitolerance and multidrug efflux functions has been noted before for MdfA and TetL [9,44], and the results of our whole cell EtBr efflux assays (Figure 5) suggest the same linkage exists in MdtM.

## Conclusions

The work presented here underlines the astonishing versatility of multidrug resistance proteins of the MFS and provides additional evidence that the multidrug efflux activity of these transporters is probably a co-opted adaptation of their original physiological function(s), thereby offering an explanation as to why these proteins persist in bacterial genomes in the absence of a selective pressure from drugs. Close homologues of MdtM are present in many pathogenic bacterial species [24] and we contend that, in all likelihood, those homologues also play a role in pH homeostasis via a monovalent metal cation/H^+^ antiport mechanism. Furthermore, we postulate that yet other MFS multidrug transporters contribute to pH homeostasis in *E. coli* and that an approach similar to the one employed here may be used to unmask them.

## Methods

All growth media, antibiotics and chemicals were purchased from Sigma-Aldrich (Poole, Dorset, UK) unless stated otherwise.

### Bacterial strains and plasmids

*E. coli* BW25113 [45] and its Δ*mdtM* and Δ*mdfA* deletion mutants [46] were obtained from the Keio collection (National BioResource Project, Japan) and used for growth assays. The Δ*mdtM* and Δ*mdfA* deletion mutants were used as the background strains for testing alkalitolerance of cells expressing wild-type *mdtM* (pMdtM) or dysfunctional MdtM D22A (pD22A) mutant from pBAD/*Myc*-His A vector (Invitrogen). Construction of these plasmids was described before [24]. The outer membrane permeability mutant *E. coli* UTL2 [47] was used for whole cell EtBr efflux assays. *E. coli* TO114 [26], a strain deficient in the Na^+^/H^+^ antiporters NhaA and NhaB, and the K^+^/H^+^ antiporter ChaA, was complemented with pMdtM or pD22A and used for production of inverted vesicles for use in transport assays.

### Bacterial growth assays on solid medium

Cultures from single bacterial colonies were grown at 37°C to an OD_600_ of 1.0 in liquid Luria Bertani (LB) medium alone (for wild-type *E. coli* BW25113), or LB media supplemented with either 30 μg/ml kanamycin (for selection of the chromosomal *mdtM*-deletion strain), or 30 μg/ml kanamycin and 100 μg/ml carbenicillin (Carbenicillin Direct, UK) (for the Δ*mdtM* BW25113 strain harboring pMdtM or pD22A). Aliquots (4 μl) from a 10^-3^ to 10^-5^ logarithmic dilution series of each culture were spotted onto plates layered with LB-agar (1% w/v tryptone, 0.5% w/v yeast extract, 1% w/v NaCl and 1.5% w/v agar). For assays performed with pMdtM and pD22A transformants the LB-agar was supplemented with the appropriate antibiotics and L-arabinose was added to a final concentration of 0.002% (w/v) to induce expression of the recombinant protein. For all the plate assays, pH of the medium was buffered by 70 mM 1,3-bis[tris(hydroxymethyl)-methylamino] propane (BTP) and pH was adjusted by HCl. Plates were incubated for 24 h at 37°C prior to imaging.

### Bacterial growth assays in liquid medium

A swab of colonies from overnight LB agar plates was used to inoculate 2 ml of LB broth containing the appropriate antibiotic(s) and, where appropriate, 0.002% (w/v) L-arabinose, and grown for 2 h with shaking at 37°C. Cultures were then diluted to an OD_600_ of 0.02 into 50 ml of fresh LB medium containing the appropriate antibiotic(s) and L-arabinose (0.002% w/v). Media were buffered by 70 mM BTP and pH was adjusted with HCl. Cells were then grown aerobically at 37°C with shaking and the OD_600_ measured every hour for 15 hours.

Assays designed to test the effects of Na^+^ or K^+^ ions at alkaline pH on the growth of BW25113 Δ*mdtM* cells transformed with pMdtM were performed in salt-free LB medium (1% w/v tryptone, 0.5% w/v yeast extract) buffered to the indicated pH with 70 mM BTP. It should be noted that although this medium is described as salt-free, residual amounts of Na^+^ and K^+^ ions are likely to be present [12,26]. Cells from the 2 ml cultures that were grown for 2 h were subsequently washed three times in the salt-free medium prior to being diluted into 50 ml of fresh salt-free medium containing 30 μg/ml kanamycin, 100 μg/ml carbenicillin, and 0.002% (w/v) L-arabinose. The media contained either no additional NaCl or KCl, or were supplemented with 20 mM, 40 mM or 86 mM NaCl or KCl. Cells were grown at 37°C with shaking and the OD_600_ measured every hour for 15 hours.

Sodium gluconate or potassium gluconate replaced NaCl or KCl, respectively, for assays designed to test for Cl^-^ ion dependence of alkalitolerance. Choline chloride or sucrose replaced the chloride salts of sodium and potassium to test for any potential osmoregulatory role for MdtM at alkaline pH. The assays were performed as described above in salt-free medium buffered to pH 9.5 with 70 mM BTP. For all assays performed in liquid medium, the pH of the cultures was measured every 5 h using a sterile glass electrode to monitor for acidification.

### Whole cell EtBr efflux assays

These assays were performed on outer membrane permeability mutant *E. coli* UTL2 cells transformed with pMdtM as described previously [24], except that 20, 50 and 100 mM NaCl was added to the loading buffer and the reaction mixture to examine the effect of Na^+^ ions on MdtM-mediated EtBr efflux activity. To ensure that Cl^-^ anions were not responsible for inhibition of EtBr efflux, 100 mM choline chloride replaced NaCl in the loading buffer and the reaction mixture. As a negative control, the EtBr efflux activity of UTL2 cells transformed with pD22A was measured.

### Measurement of transmembrane ΔpH

Assays of K^+^/H^+^ and Na^+^/H^+^ antiport were based on those described in [48] and were conducted by measuring the fluorescence quenching /dequenching of the pH-sensitive indicator acridine orange upon addition of the test cations to energized inverted membrane vesicles generated from antiporter-deficient *E. coli* TO114 cells that overproduced recombinant wild-type MdtM. Control experiments were performed on inverted vesicles generated from TO114 cells that overproduced dysfunctional MdtM from pD22A.

Cells were grown and inverted vesicles were generated using the protocols described in [25]. The total membrane protein concentration of the vesicles was determined using the bicinchoninic acid assay (Thermo Scientific Pierce, Rockford, IL) according to the manufacturer’s protocol. Transport measurements were performed at the indicated pH values (ranging between pH 6.5 to 9.75) at 25°C using a Fluoromax-4 fluorometer (Horiba UK Ltd, Middlesex, UK). Inverted vesicles were excited at 492 nm and the fluorescence emission recorded at 525 nm. The excitation and emission slit widths were set to 1.5 nm and 2.5 nm, respectively. Inverted membrane vesicles were added to reaction buffer (10 mM BTP adjusted to the indicated pH with HCl, 5 mM MgSO_4_ and 1 μM acridine orange) in a quartz cuvette to a final concentration of 0.5 mg/ml membrane protein in a total volume of 1.5 ml. For each assay, the inverted vesicle mixture was allowed to equilibrate for ~300 s prior to recording of the fluorescence signal. To initiate respiration-dependent generation of ΔpH (acid inside), a final concentration of 2 mM Tris-D-L-lactate, made up in reaction buffer at the desired pH, was added to the reaction mixture at the time indicated. Once a stable ΔpH was established, and the fluorescence quench of acridine orange reached steady state (usually after ~200 s), sodium gluconate or potassium gluconate at a final concentration of 100 mM was added to assess the ability of external K^+^ and Na^+^ to act as substrates for antiport with internal H^+^. Gluconate rather than chloride salts of the metal cations were used to avoid any potential interference with the assay by Cl^-^ ions [49]. The fluorescence dequenching upon addition of Na^+^ or K^+^ (due to dissipation of the established ΔpH as a result of MdtM-mediated metal cation/H^+^ antiport activity) was monitored for an additional 60 s prior to the addition of 100 μM of the protonophore carbonyl cyanide 3-chlorophenylhydrazone (CCCP) to completely dissipate the ΔpH and abolish transport. All experiments were performed in triplicate on at least two separate preparations of inverted vesicles.

The results of the transport assays were used to construct a pH profile of transport activity as described in [42]. Briefly, MdtM-mediated Na^+^/H^+^ and K^+^/H^+^ antiport activity at every pH value tested was calculated as the percent dequenching of the acridine orange fluorescence relative to the initial respiration-dependent quench. The calculated activities were corrected for nonspecific background activity by subtraction of the dequenching measured in the comparative controls.

### Assessment of the apparent affinity of MdtM for Na^+^ and K^+^ cations

The affinity of MdtM for transported Na^+^ and K^+^ ions was estimated by measuring the concentration of each ion that was required to elicit the half-maximal, steady-state percent dequenching of acridine orange fluorescence in inverted vesicles derived from TO114 cells transformed with pMdtM. The fluorescence dequench response was initiated by addition of varying concentrations (from 5 mM to 125 mM) of cation to the inverted vesicles as described before [42,50-52]. Fluorescence-based assays of the Na^+^/H^+^ and K^+^/H^+^ activity of MdtM in *E. coli* TO114 inverted vesicles were conducted over a range of concentrations of added Na^+^ gluconate or K^+^ gluconate. The assays were performed at 25°C at the previously determined pH optimum for each antiport reaction (pH 9.25 and pH 9.0 for Na^+^/H^+^ and K^+^/H^+^, respectively); the activity observed in inverted vesicles from the pD22A control transformant was subtracted from the recombinant wild-type MdtM activity at each substrate concentration to obtain the values shown. The [Na^+^]_1/2_ and [K^+^]_1/2_ values were determined from nonlinear regression analysis of the data using SigmaPlot 10 (Systat, Richmond, CA).

### Measurement of transmembrane Δψ

The Δψ-sensitive fluorophore Oxonol V [bis-(3-phenyl-5-oxoisoxazol-4-yl)pentamethine oxonol] (Cambridge Bioscience Ltd, Cambridge, UK) was used to determine if the MdtM-mediated antiport observed in the previous experiments was electrogenic. Inverted vesicles were produced from TO114 cells transformed with pMdtM or pD22A as described previously [25], except that the vesicle resuspension buffer was made Cl^-^-free by substitution of the 140 mM choline chloride component with 280 mM sorbitol [42] and by using H_2_SO_4_ rather than HCl to adjust buffer pH. Inverted vesicles produced from *E. coli* BW25113 cells that retained the full complement of electrogenic Na^+^/H^+^ antiporters provided a positive control. Vesicles (500 μg/ml membrane protein) were added to assay buffer (10 mM BTP, 5 mM MgSO_4_, 5 μM Oxonol V) that had its pH adjusted to 9.0 (for detection of electrogenic K^+^/H^+^ antiport) or 9.25 (for detection of electrogenic Na^+^/H^+^ antiport). The pH of the assay buffer used for positive control BW25113 vesicles was adjusted to 8.5 to ensure detection of electrogenic NhaA-catalysed Na^+^/H^+^ antiport activity [30]. All vesicles were incubated on ice for 200 s prior to addition of 2 mM Tris-D-L-lactate to initiate respiration-dependent generation of Δψ, and the resultant quenching of Oxonol V fluorescence was monitored at 25°C using a Fluoromax-4 fluorometer with an excitation wavelength of 599 nm and emission wavelength of 634 nm. Excitation and emission slit widths were set to 10 nm and 20 nm, respectively. Electrogenic antiport activity was estimated on the basis of its ability to dissipate the established Δψ (recorded as a dequenching of the fluorescence signal) in response to addition of 100 mM Na^+^ gluconate or K^+^ gluconate to vesicles at the times indicated. Addition of 100 μM CCCP was used to abolish both Δψ and ΔpH components of the PMF. As a further control, 1 μM of the ionophore nigericin (which at low concentrations selectively consumes ΔpH in the presence of K^+^ via electroneutral K^+^/H^+^ exchange)[5] was added to vesicles of TO114 cells transformed with pMdtM. These vesicles were incubated in assay buffer that contained 50 mM K^+^ gluconate, and valinomycin (5 μM) was added to selectively abolish Δψ.

### Measurement of cytoplasmic pH

The intracellular pH of *E. coli* whole-cell suspensions at various external alkaline pH values was determined by ratiometric fluorescence measurements of the acetoxymethyl ester derivative of the membrane-permeant, pH-sensitive fluorescent probe, 2,7-bis-(2-carboxyethyl)-5-carboxyfluorescein (BCECF-AM; Life Technologies Ltd, Paisley, UK) [53]. Intracellular pH was correlated to fluorescence signal by recording the fluorescence emission intensity of BCECF at 530 nm upon excitation at 490 nm (the BCECF pH-dependent excitation wavelength) and at 440 nm (the BCECF pH-independent excitation wavelength). Calculation of the ratio intensities at the two excitation wavelengths permitted intracellular pH measurements that were independent of intensity losses due to changes in the absorption profile of the probe and/or photobleaching.

Calibration of the system was performed on suspensions of *E. coli* Δ*mdtM* BW25113 cells. Cultures from single bacterial colonies were grown aerobically at 30°C to an OD_600_ of 3.0 in LB medium supplemented with 30 μg/ml kanamycin. Cultures were then diluted 125-fold into 100 ml of fresh LB medium containing antibiotic and grown aerobically at 37°C to an OD_600_ of 1.0. Six 10 ml aliquots of cells were pelleted by centrifugation (3000 × *g*) at 4°C and washed twice in assay buffer (140 mM NaCl, 10 mM HEPES and 1 mM MgCl_2_) that had pH adjusted with KOH to 7.5, 8.0, 8.5, 9.0, 9.25 or 9.5. To load the cells with fluorescent probe, the washed cells were pelleted and then resuspended to OD_600_ of 2.0 in assay buffer that contained 2.5 μM BCECF-AM. To equalize internal and external pH, 10 μM of the protonophore CCCP was added to the buffer and the cells were incubated in the dark at 37°C for 1 h. BCECF-AM-loaded cells were collected by centrifugation and stored on ice until use. For each pH value investigated, 200 μl of loaded cells were added to 1.3 ml of assay buffer that contained 10 μM CCCP. After incubation at 30°C for 60 s, the fluorescence intensity of the mixture at 530 nm upon excitation at 490 nm and 440 nm was recorded under continuous stirring using a Fluoromax-4 fluorometer with excitation and emission slit widths set to 1.0 nm and 10 nm, respectively. Experiments were performed in triplicate for each pH value investigated and used to construct a calibration plot that correlated the 490 nm/440 nm fluorescence emission ratio to pH.

To determine if MdtM functioned in maintenance of a stable intracellular pH under conditions of alkaline stress, fluorescence measurements were performed on pMdtM and pD22A transformants of *E. coli* Δ*mdtM* BW25113 cells at six different external alkaline pH values using the method described above except that carbenicillin (100 μg/ml) and L–arabinose (0.002% w/v) was added to the growth medium, CCCP was omitted from the assay buffer, and D-glucose (1 mM) was added to the assay buffer to energise the cells 60 s prior to recording the fluorescence.

### Western blot analysis of recombinant MdtM

Estimation of expression levels of recombinant wild-type and D22A mutant MdtM by transformed Δ*mdtM* BW25113 cells grown at different alkaline pH values was performed as described in [25].

## Competing interests

The authors declare no competing interests.

## Authors’ contributions

SRH performed the experimental work described in the study and participated in its design. CJL conceived of, designed and coordinated the study, and wrote the manuscript. Both authors read and approved the final manuscript.

## Supplementary Material

Additional file 1 **PDF file showing that *E. coli ***Δ ***mdfA *cells complemented with plasmidic wild-type *mdtM *can grow at alkaline pH. Growth of Δ *mdfA E. coli *BW25113 cells complemented with pMdtM or the pD22A mutant in liquid LB media at different alkaline pH values.** Data points and error bars represent the mean ± SE of three independent measurements. Click here for file
