# Erratum to: Multidrug resistance protein MdtM adds to the repertoire of antiporters involved in alkaline pH homeostasis in *Escherichia coli*

**DOI:** 10.1186/s12866-017-0995-5

**Published:** 2017-04-12

**Authors:** Scarlett R. Holdsworth, Christopher J. Law

**Affiliations:** Institute for Global Food Security, School of Biological Sciences, Medical Biology Centre, Queen’s University Belfast, Belfast, UK

## Erratum

It has been brought to our attention after publication that there is an error in Fig. 2 of our article [1]. The panel in Fig. 2a which purported to show the growth phenotypes of E.coli BW25113 cells transformed with plasmids pMdtM and pD22A at logarithmic dilutions of 10^–3^ to 10^–5^ at pH 9.75 actually showed the 10^–4^ to 10^–6^ logarithmic dilutions of cells grown at pH 9.5. The offending panel has been removed and the correct panel, which shows the growth phenotypes of transformants at logarithmic dilutions of 10^–3^ to 10^–5^ at pH 9.75, has been incorporated into the new Fig. 2 shown at the end of this erratum. The figure legend remains unchanged. This error does not affect the other results or any of the conclusions of the article.

**Fig. 2:**
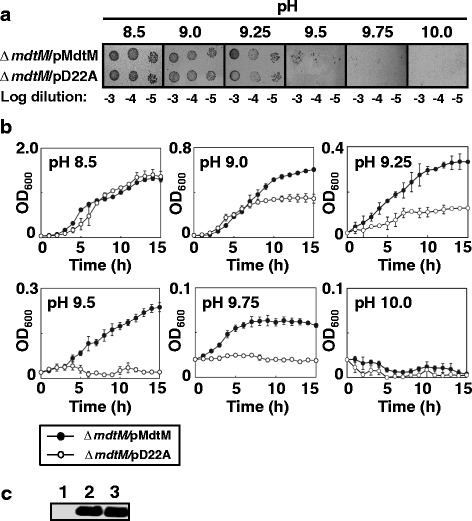
*E. coli* Δ*mdtM* cells complemented with wild-type *mdtM* can grow at alkaline pH. **a** Growth phenotypes of Δ*mdtM E. coli* BW25113 cells transformed with a multicopy plasmid encoding wild-type MdtM (pMdtM) or the dysfunctional MdtM D22A mutant (pD22A) at different alkaline pH’s on LB agar. As indicated, 4 μl aliquots of a logarithmic dilution series of cells were spotted onto the solid media and the plates were incubated for 24 h at 37 °C prior to digital imaging. **b** Growth of Δ*mdtM E. coli* BW25113 cells complemented with pMdtM or the pD22A mutant in liquid LB media at different alkaline pH values. Data points and error bars represent the mean ± SE of three independent measurements. **c** Comparison of expression levels of recombinant wild-type and D22A mutant MdtM at three different pH values by Western blot analysis of DDM detergent-solubilised membranes of *E. coli* BW25113 cells that overproduced the protein from plasmidic DNA. Cells harbouring empty pBAD vector were used as a negative control. Each lane contained 10 μg of membrane protein
