# Supplementary figures and images for: Multidrug resistance protein MdtM adds to the repertoire of antiporters involved in alkaline pH homeostasis in Escherichia coli
Source: BMC Microbiol. 2013 May 23;13:113. doi: 10.1186/1471-2180-13-113 (PMC3668916; doi:10.1186/1471-2180-13-113)

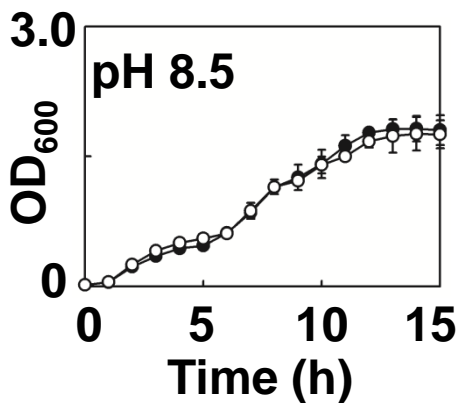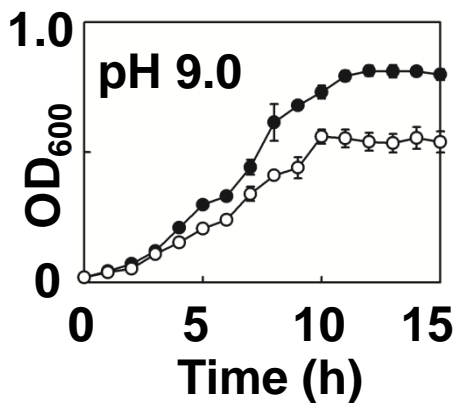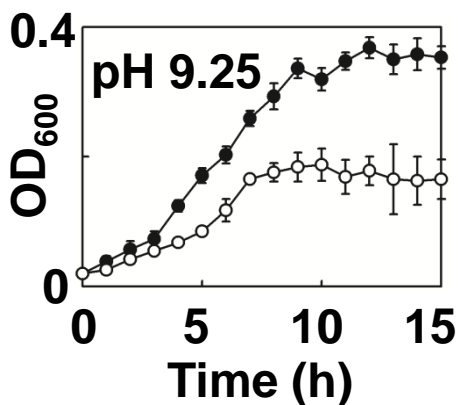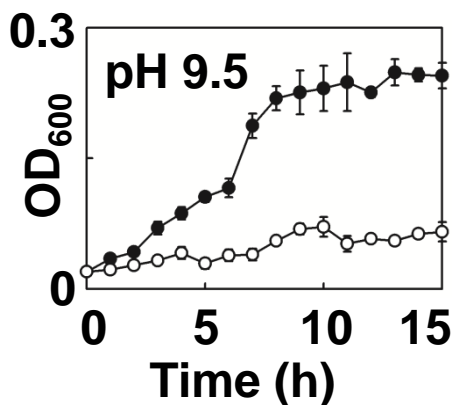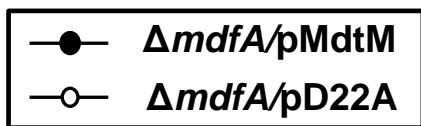

Supplement: Additional file 1 — PDF file showing that E. coli Δ mdfA cells complemented with plasmidic wild-type mdtM can grow at alkaline pH. Growth of Δ mdfA E. coli BW25113 cells complemented with pMdtM or the pD22A mutant in liquid LB media at different alkaline pH values. Data points and error bars represent the mean ± SE of three independent measurements. [file 1471-2180-13-113-S1.pdf]
